# Adverse clinical outcomes associated with a low dose and a high dose of aspirin following percutaneous coronary intervention: a systematic review and meta-analysis

**DOI:** 10.1186/s12872-016-0347-7

**Published:** 2016-09-02

**Authors:** Pravesh Kumar Bundhun, Girish Janoo, Abhishek Rishikesh Teeluck, Wei-Qiang Huang

**Affiliations:** 1 Institute of Cardiovascular Diseases, the First Affiliated Hospital of Guangxi Medical University, Nanning, Guangxi 530027 People’s Republic of China; 2 Guangxi Medical University, Nanning, Guangxi 530027 People’s Republic of China

**Keywords:** Aspirin, Percutaneous coronary intervention, Bleeding, Major adverse cardiac events, Cardiovascular death, Meta-analysis

## Abstract

**Background:**

Guidelines from the American Heart Association/American College of Cardiology recommend a higher dosage of aspirin daily following Percutaneous Coronary Intervention (PCI), whereas guidelines from the European Society of Cardiology recommend a lower dosage. This study aimed to compare the adverse clinical outcomes associated with a low dose and a high dose of aspirin following PCI.

**Methods:**

Electronic databases were searched for studies comparing a low dose with a high dose aspirin following PCI. Adverse clinical outcomes were considered as the endpoints in this study. We calculated Odds Ratios (OR) with 95 % Confidence Intervals (CIs) for categorical variables. The pooled analyses were performed with RevMan 5.3 software.

**Results:**

A total number of 25,083 patients were included. Results from this analysis showed that the combination of Cardiovascular (CV) death/Myocardial Infarction (MI) or stroke was not significantly different between a low and high dose of aspirin with OR: 1.08, 95 % CI: 0.98–1.18; *P* = 0.11. Mortality and MI were also not significantly different between these two treatment regimens following PCI with OR: 0.95, 95 % CI: 0.74–1.23; *P* = 0.71 and OR: 1.17, 95 % CI: 0.97–1.41; *P* = 0.09 respectively. However, a high dose of aspirin was associated with a significantly higher rate of Major Adverse Cardiac Events (MACEs) with OR: 1.20, 95 % CI: 1.02–1.41; *P* = 0.03. Thrombolysis In Myocardial Infarction (TIMI) defined minor bleeding was also significantly higher with a high dose aspirin with OR: 1.22, 95 % CI: 1.02–1.47; *P* = 0.03. When Stent thrombosis (ST) was compared, no significant difference was found with OR: 1.28, 95 % CI: 0.59–2.58; *P* = 0.53. Even if TIMI defined major bleeding favored a low dose of aspirin, with OR: 1.42, 95 % CI: 0.95–2.13; *P* = 0.09, or even if major bleeding was insignificantly higher with a high dose aspirin, with OR: 1.78, 95 % CI: 1.01–3.13; *P* = 0.05; I^2^ = 94 %, higher levels of heterogeneity observed in these subgroups could not be considered significant to any extent.

**Conclusion:**

According to the results of this analysis, a high dose of aspirin following PCI was not associated with any significantly higher rate of CV death/MI/stroke, mortality or MI. However, MACEs significantly favored a low dose of aspirin. In addition, TIMI defined minor bleeding was significantly higher with a high dose of aspirin whereas the results for the major bleeding outcomes were not statistically significant. However, due to limited data availability and since the subgroups analyzing major bleeding were highly heterogeneous, further studies are recommended to completely solve this issue.

## Background

Percutaneous Coronary Intervention (PCI) is considered to be among the most preferred invasive procedures carried out in patients with Acute Coronary Syndrome (ACS). Dual Anti-Platelet Therapy (DAPT) with aspirin and a P2Y12 inhibitor mainly clopidogrel, showed increased benefits in reducing adverse clinical outcomes following PCI with Drug Eluting Stents (DES) or Bare Metal Stents (BMS) [1]. Therefore, the American College of Cardiology/American Heart Association [2] recommends at least one-year treatment with DAPT after PCI with DES whereas the European Society of Cardiology [3] recommends 6 to 12 months DAPT use after intracoronary stenting by DES. For BMS, the duration period for DAPT is even shorter (1 month) compared to DES. However, uncertainty regarding the optimal dosage of aspirin is still a fact which remains to be solved [4]. Guidelines from the American Heart Association/American College of Cardiology recommend higher doses of aspirin (162 to 325 mg) daily following PCI [5], whereas guidelines from the European Society of Cardiology recommend lower doses (75 to 100 mg) [6]. This current analysis aimed to compare the adverse clinical outcomes associated with a low dose and a high dose of aspirin in patients with ACS following PCI with either DES or BMS.

## Methods

### Data sources and search strategy

The Cochrane Library, PubMed, Medline and EMBASE were searched for studies comparing a low dose with a high dose of aspirin following PCI by typing the words or phrases ‘low and high dose aspirin and percutaneous coronary intervention’. Another search was conducted using the words ‘aspirin and acute coronary syndrome or drug eluting stents/bare metal stents’ [aspirin + acute coronary syndrome/aspirin + percutaneous coronary intervention/aspirin + drug eluting stents/bare metal stents/low and/or high dose aspirin + percutaneous coronary intervention]. In order to enhance this search, abbreviations such as ASA, ACS, DES/BMS and PCI were also used as well as the terms ‘coronary angioplasty’, ‘coronary intervention’ and ‘single or double dose aspirin’ [ASA + PCI/ASA + percutaneous coronary intervention/ASA + acute coronary syndrome/ASA + ACS/ASA + DES/BMS/ASA + coronary angioplasty]. Medical journals which were expected to publish articles related to coronary interventions such as the Journal of Circulation, the Journal of the American College of Cardiology, Euro-intervention, the American Journal of Cardiology and BMC cardiovascular disorders were also searched using the above mentioned terms for relevant articles. Moreover, reference lists of suitable articles were also searched for relevant studies. This search was restricted to articles published in English.

### Inclusion and exclusion criteria

Studies were included if:

- They were Randomized Controlled Trials (RCTs) or observational studies comparing a low dose with a high dose of aspirin following PCI.
- They reported adverse outcomes as their clinical endpoints.
- They involved any dosage of aspirin, as far as a low dose was compared with a high dose.

Studies were excluded if:

- They were meta-analyses, letter to editors and case studies.
- They did not report adverse outcomes as their clinical endpoints.
- They were duplicates.

### Outcomes, definitions and follow ups

The clinical endpoints analyzed included:

- Mortality
- Myocardial Infarction (MI)
- Cardiovascular (CV) death/MI/stroke
- Major adverse cardiac events (MACEs) consisting of death, MI and revascularization
- Stent Thrombosis (ST)
- Major bleeding which was defined as bleeding that was significantly disabling for example intraocular bleeding that lead to significant vision loss, or bleeding requiring transfusion of 2 units of red blood cells or equivalent whole blood, a drop in hemoglobin concentration of 5 g/L, bleeding causing significant hypotension requiring intravenous inotropes or surgical intervention, symptomatic intracranial hemorrhage or bleeding that was fatal
- TIMI defined major bleeding [7]
- TIMI defined minor bleeding

The outcomes reported and the dosage of aspirin reported among the cohorts as well as their corresponding follow up periods have been summarized in Table 1.

**Table 1:** Reported outcomes

Studies	Outcomes reported	Dosage of aspirin	Follow up periods
GHOST	MACEs, ST, Death or MI, TIMI bleeding	81 mg vs 160–325 mg	1 year
CURRENT OASIS 7	Death/MI/stroke, death, MI, stroke, TIMI major and minor bleeding	≤100 mg vs ≥ 300 mg	30 days
CURE	CV death/MI/stroke, major bleeding	<200 mg vs ≥ 200 mg	1 year
HORIZONS-AMI	MACEs, mortality, MI, stroke, major bleeding, TIMI major and minor bleeding, ST	≤200 mg vs > 200 mg	3 years

*Abbreviations*: *MI* myocardial infarction, *TIMI* thrombolysis in myocardial infarction, *MACEs* major adverse cardiac events, *ST* stent thrombosis, *CV* cardiovascular

A low dosage of aspirin was defined as any low dosage in accordance to the high dosage of aspirin reported in the same study. For example, if a dosage of aspirin greater than 200 mg was considered a high dosage, then any dosage below 200 mg in the same study should be considered as a low dosage.

### Data extraction and quality assessment

Three authors (PKB, GJ and ART) independently assessed the articles selected for this analysis. Information concerning the type of study reported, the total number of patients treated with a low and high dose of aspirin respectively, data concerning the baseline characteristics of the patients, the reported outcomes and follow up periods were carefully extracted. If any disagreement about including certain data occurred, it was discussed among these three authors and if they could not reach a consensus, a final decision was made by the fourth author (WQH). Bias risk was assessed in accordance to the components recommended by the Cochrane Collaboration [8].

### Methodological quality and statistical analysis

Recommendations of the PRISMA (Preferred Reporting Items for Systematic Reviews and Meta-Analyses) statement were followed since this is a meta-analysis involving mostly trials [9]. Heterogeneity across the subgroups was assessed using the Cochrane Q-statistic test (whereby a *P* value < 0 · 05 was considered statistically significant whereas a *P* value > 0.05 was considered statistically insignificant) and the I^2^-statistic test (whereby an I^2^ with low percentage represented a lower heterogeneity and an increasing percentage denoted an increasing heterogeneity). If I^2^ was less than 50 %, a fixed effect model was used. However, if I^2^ was more than 50 %, a random effect model was used. Publication bias was visually estimated by assessing funnel plots. We calculated Odds Ratios (OR) and 95 % Confidence Intervals (CIs) for categorical variables. The pooled analyses were performed with RevMan 5.3 software. Ethical approval was not required for systematic reviews and meta-analyses. All the authors had full access to the data and approved the manuscript as written.

## Results

### Search results

**Fig. 1:**
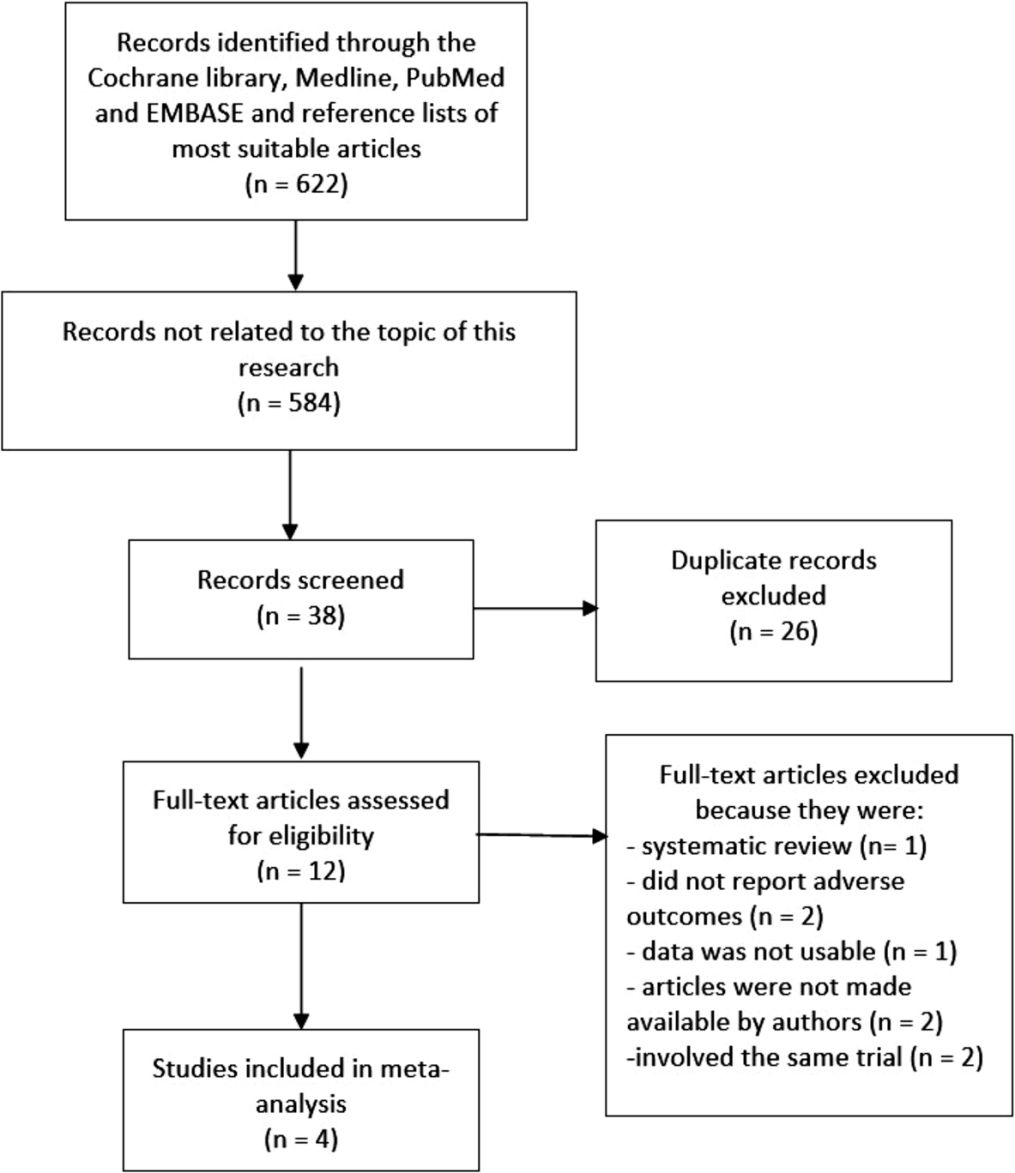
Flow diagram representing the study selection

A total number of 622 articles were obtained from the Cochrane Library, PubMed, Medline and EMBASE and from reference lists of suitable articles. After a careful assessment of titles and abstracts, 584 articles were eliminated since they were not related to our topic. A further 26 articles were eliminated since they were duplicates. 12 full-text articles were assessed for eligibility. Eight more articles were eliminated: one article was a systematic review of the literature, two article did not report adverse clinical outcomes and two articles which could probably satisfy the inclusion and exclusion criteria of our study were not made available by the authors, one article did not include data which could be used in this analysis, and another two trials were the subset of other trials included in this analysis. Finally, four articles (3 trials and 1 observational study) [4, 10–13] were included in this systematic review and meta-analysis. The flow diagram for the study selection has been represented in Fig. 1.

### General features of the studies included

The general features of the studies included in this meta-analysis have been listed in Table 2.

**Table 2:** General features of the studies included

Studies	Type of study	No of patients with low dose ASA (n)	No of patients with high dose ASA (n)	Total no of patients (n)	Type of P2Y12 inhibitor used
GHOST [10]	observational	313	2507	2820	clopidogrel
CURRENT OASIS 7 [11]	RCT	3371	3502	6873	clopidogrel
CURE [4, 12]	RCT	8429	4110	12,539	clopidogrel
HORIZONS-AMI [13]	RCT	2289	562	2851	clopidogrel
Total no of patients (n)		14,402	10,681	25,083	

Only female patients were included from trial CURRENT OASIS 7 in order to avoid the influence of this trial on the results of this analysis

*Abbreviations*: *ASA* aspirin, *RCT* randomized controlled trials

A total number of 25,083 patients (14,402 patients were assigned to a low dose of aspirin and 10,681 patients were assigned to a high dose of aspirin) were included in this analysis.

### Baseline characteristics of the studies included

The baseline features of the patients have been listed in Table 3 whereas Table 4 shows the other antiplatelet/anticoagulants used by the patients during the procedure or following PCI. According to these baseline features, no significant differences were observed between patients assigned to a low dose and a high dose of aspirin respectively.

**Table 3:** Baseline features of the patients included in this analysis

Studies	Mean age (years)	Males (%)	Ht (%)	Ds (%)	Cs (%)	DM (%)
	L/H	L/H	L/H	L/H	L/H	L/H
GHOST	67.0/64.0	64.0/70.0	73.0/65.0	76.0/72.0	19.0/27.0	12.0/8.0
CURRENT OASIS 7	61.2/61.5	0.00/0.00	60.2/60.4	40.9/41.4	33.6/33.2	23.1/23.8
CURE	-	58.8/65.4	58.8/60.5	-	20.8/25.1	21.0/26.8
HORIZONS-AMI	59.9/58.8	76.3/79.4	50.6/58.2	42.3/47.9	65.0/63.5	16.6/15.8

*Abbreviations*: *L* low dose, *H* high dose, *Ht* hypertension, *Ds* dyslipidemia, *Cs* current smoker, *DM* diabetes mellitus

**Table 4:** Other antiplatelet/anticoagulants used by the patients included in this analysis

Other antiplatelets/anticoagulants	GHOST	CURRENT OASIS 7	CURE	HORIZONS-AMI
Heparin	+++	−	−	−
GP IIb/IIIa inhibitors	+	+	+++	−
Oral anticoagulants (warfarin/Coumadin)	+	−	+	+
Clopidogrel	++++	++++	++++	++++

*Abbreviations*: *GP* glycoproteins, “+”: less than 25 % of patients, “++”: 26 to 50 % of patients, “+++”: 51 to 75 % of patients, “++++”: 76 to 100 % of patients

### Clinical outcomes reported

**Fig. 2:**
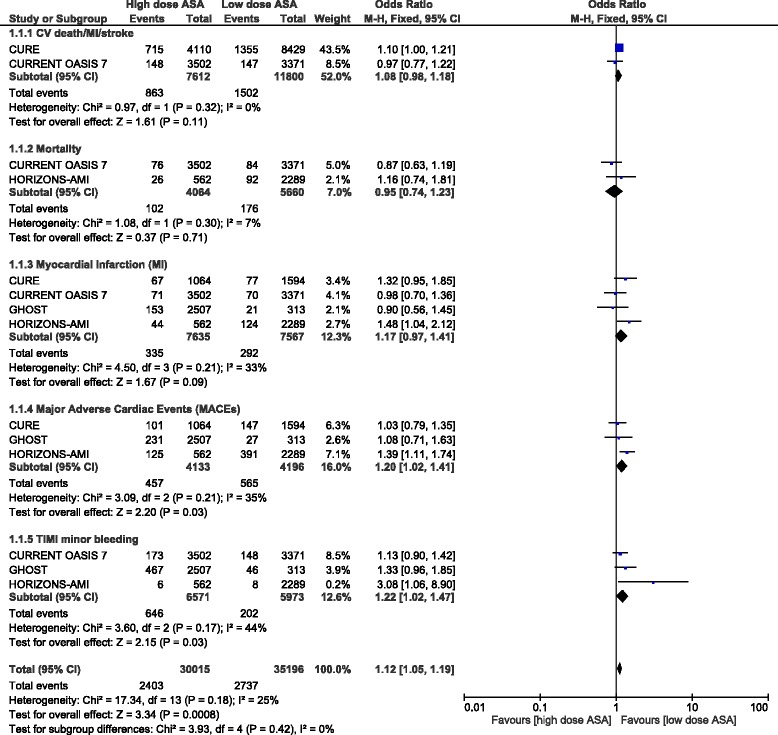
Adverse clinical outcomes reported between a low and a high dose of aspirin

Results from this analysis (Table 5) showed that the combination of CV death/MI or stroke was not significantly different between a low and a high dose of aspirin following PCI with OR: 1.08, 95 % CI: 0.98–1.18; *P* = 0.11, I^2^ = 0 %. Mortality and MI were also not significantly different between these two treatment regimens after PCI with OR: 0.95, 95 % CI: 0.74–1.23; *P* = 0.71, I^2^ = 7 % and OR: 1.17, 95 % CI: 0.97–1.41; *P* = 0.09, I^2^ = 33 % respectively. However, a high dose of aspirin was associated with a significantly higher rate of MACEs with OR: 1.20, 95 % CI: 1.02–1.41; *P* = 0.03, I^2^ = 35 %. TIMI defined minor bleeding was also significantly higher with a high dose aspirin with OR: 1.22, 95 % CI: 1.02–1.47; *P* = 0.03; I^2^ = 44 %. These results have been represented in Fig. 2.

**Table 5:** Results of this analysis

Outcomes analyzed	OR with 95 % CI	*P* value	I^2^ (%)
Mortality	0.95 [0.74–1.23]	0.71	7
MI	1.17 [0.97–1.41]	0.09	33
CV death/MI/stroke	1.08 [0.98–1.18]	0.11	0
MACEs	1.20 [1.02–1.41]	0.03	35
ST	1.28 [0.59–2.78]	0.53	65
Major bleeding	1.78 [1.01–3.13]	0.05	94
TIMI major bleeding	1.42 [0.95–2.13]	0.09	59
TIMI minor bleeding	1.22 [1.02–1.47]	0.03	44

*Abbreviations*: *MI* myocardial infarction, *TIMI* thrombolysis in myocardial infarction, *MACEs* major adverse cardiac events, *ST* stent thrombosis, *CV* cardiovascular, *OR* odds ratio, *CI* confidence intervals

**Fig. 3:**
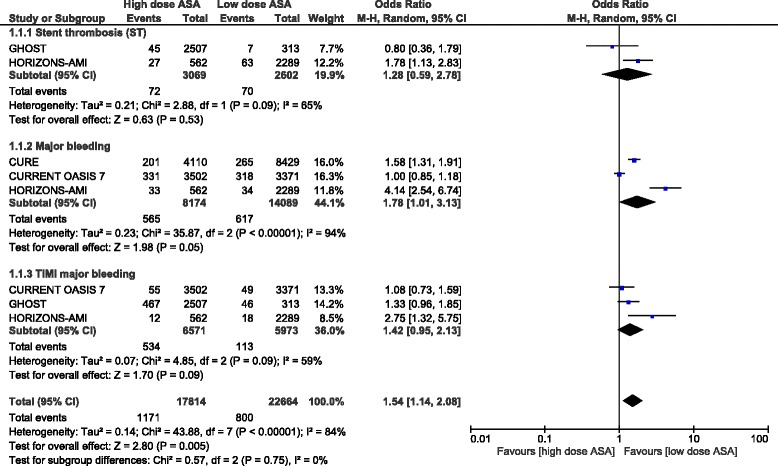
Stent thrombosis, major bleeding outcomes reported between a low and a high dose of aspirin

When ST was compared between these two groups, no significant difference was found with OR: 1.28, 95 % CI: 0.59–2.58; *P* = 0.53, I^2^ = 65 %. Even if TIMI defined major bleeding favored a low dose aspirin, with OR: 1.42, 95 % CI: 0.95–2.13; *P* = 0.09; I2 = 59 %, the result was not statistically significant. Moreover, even if major bleeding was higher with a high dose aspirin, with OR: 1.78, 95 % CI: 1.01–3.13; *P* = 0.05; I^2^ = 94 %, the level of heterogeneity was much higher that it could not be considered significant to any extent. These results have been represented in Fig. 3.

**Fig. 4:**
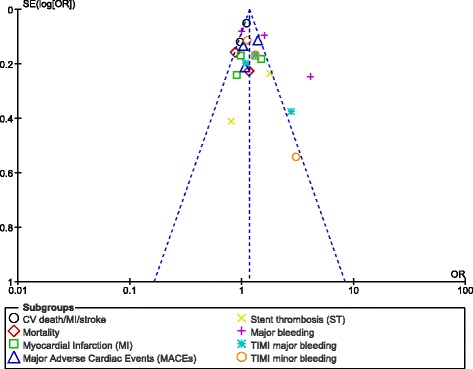
Funnel plot showing sensitivity analysis

For all of the above analyses, sensitivity analyses yielded consistent results. Based on a visual inspection of the funnel plot obtained, there has been little evidence of publication bias for the included studies that assessed several clinical endpoints. However, a high level of heterogeneity was observed among the subgroups analyzing stent thrombosis and the major bleeding outcomes. The funnel plot showing the sensitivity analysis has been represented in Fig. 4.

## Discussion

This study aimed to compare the adverse clinical outcomes associated with a low dose and a high dose of aspirin following PCI. Results of this study showed that a high dose of aspirin was not associated with a significantly higher rate of mortality, CV death/MI/stroke and MI. ST was also not significantly different between these two dosages of aspirin. However, MACEs significantly favored a low dose aspirin. In addition, a high dose of aspirin was associated with a significantly higher rate of TIMI defined minor bleeding, without any significant increase in TIMI defined major bleeding major bleeding after PCI.

The systematic review of literature [14] which was meant to show any association between aspirin dosing and cardiac and bleeding events after treatment of ACS showed no improved clinical outcomes associated with a high dose of aspirin following PCI among the 289,330 patients analyzed. 2.1 % of patients experienced major bleeding when treated with a high dose of aspirin whereas only 1.9 % of patients treated with a low dose of aspirin following stent implantation experienced major bleeding.

Moreover, the investigators of the CURRENT OASIS 7 [11] concluded that in patients with ACS who were referred for an invasive strategy, no significant difference in primary outcome of cardiovascular death, MI or stroke was observed between a low and a high dose of aspirin. However, only a follow up period of 7 days was considered.

Also, the study published by Joyal et al. [15] demonstrating the influence of a low dose (81 mg) versus a high dose (325 mg) of aspirin on the incidence of sirolimus eluting stents showed a similar rate of ST to be associated with either a low or a high dose of aspirin. The Ottawa Heart Institute PCI Registry [16] which involved 930 patients discharged on 325 mg aspirin and 910 patients discharged on 81 mg aspirin showed no difference in death or MI at 1 year between these two different dosages of aspirin. In addition, another study investigating the influence of low dose aspirin (81 mg) on the incidence of definite stent thrombosis in patients receiving BMS and DES concluded that a low dose of aspirin following PCI was not associated with any increase in definite stent thrombosis compared to a high dose [17].

However, results from the Dual Antiplatelet Therapy Study [18] showed that a high dose of aspirin might be associated with adverse events and the authors suggested that a low dose of aspirin might be the target to improve clinical outcomes after PCI reflecting the results of this current analysis.

Nevertheless, when prasugrel was compared with clopidogrel, with a high and low dose aspirin respectively, prasugrel was associated with better clinical outcomes irrespective of the dosage of aspirin as demonstrated in the TRITON TIMI 38 trial whereby 12,674 patients were classified into a low and high dose aspirin groups [19]. No meaningful interaction of aspirin with clopidogrel was observed. However, this current analysis was different and was focused mainly on comparing a low with a high dose of aspirin following PCI.

### Novelty

This study is new in the way that it is among the first systematic review and meta-analyses comparing a low dose with a high dose of aspirin following PCI. Moreover, several adverse outcomes have been analyzed. This study also included a large number of patients from randomized trials compared to patients from observational studies and reported a low or moderate level of heterogeneity among several subgroups assessing these clinical endpoints. Since dosage of aspirin following PCI could be an important issue in several types of patients, this analysis could inspire other scientists to conduct further research in this particular field.

### Limitations

This study also has limitations. First of all, due to the limited number of patients and studies, this analysis might not provide robust results. Secondly, because this analysis also included data obtained from observational studies along with data obtained from randomized trials, selection bias could possibly have been introduced. Moreover, one study had a follow-up period of 3 years and another one had a follow up period of 1 month. They have been included among other studies with a follow-up period of 1 year and analyzed altogether. This could be another limitation. In addition, several subgroups analyzed only compared data from two or three studies which could strictly affect the results and should be considered another limiting factor in this meta-analysis. A high level of heterogeneity observed among the several subgroups analyzing stent thrombosis, and major bleedings could be another limitation in this study. In addition, different studies reported different dosage of aspirin. This could also be a limiting factor in this study. Also, only the percentage of patients representing the female population were included from the trial CURRENT OASIS 7, because its large number of patients could influence the results of this analysis. Therefore, this could also be a limitation in this study. It should also be noted that patients involved in this analysis varied from stable coronary diseases, ST segment elevated MI or non-ST segment elevated MI representing another probable limitation to this study. However, the main focus was on patients who underwent PCI. Also, almost all of the studies were sub-group analysis of trials whereby patients were not randomized specifically to a low and high dosage of aspirin respectively, therefore, the groups might not necessarily be comparable thus rendering the introduction of potentially confounding variables possible, which could have a great impact on the results of this current analysis. Another limitation could be the influence of glycoprotein IIb/IIIa inhibitors used peri-operatively, and other anticoagulants which were used differently following PCI in different studies, possibly affecting the bleeding outcomes.

## Conclusion

According to the results of this analysis, a high dose of aspirin following PCI was not associated with any significantly higher rate of CV death/MI/stroke, mortality or MI. However, MACEs significantly favored a low dose of aspirin. In addition, TIMI defined minor bleeding was significantly higher with a high dose of aspirin whereas the results for the major bleeding outcomes were not statistically significant. However, due to limited data availability and since the subgroups analyzing major bleeding were highly heterogeneous, further studies are recommended to completely solve this issue.

## Abbreviations

ACS: Acute coronary syndrome
MACEs: Major adverse cardiac events
OR: Odds ratio
PCI: Percutaneous coronary intervention
TIMI defined bleeding: Thrombolysis in myocardial infarction defined bleeding
